# Development of a Calibration Strip for Immunochromatographic Assay Detection Systems

**DOI:** 10.3390/s16071007

**Published:** 2016-06-29

**Authors:** Yue-Ming Gao, Jian-Chong Wei, Peng-Un Mak, Mang-I. Vai, Min Du, Sio-Hang Pun

**Affiliations:** 1 College of Physics and Information Engineering, Fuzhou University, Fuzhou 350116, China; jjasonway@163.com (J.-C.W.); dm_dj90@163.com (M.D.); 2 Key Lab of Medical Instrumentation & Pharmaceutical Technology of Fujian Province, Fuzhou 350116, China; fstpum@umac.mo (P.-U.M.); fstmiv@umac.mo (M.-I.V.); 3 State Key Laboratory of Analog and Mixed Signal VLSI, University of Macau, Macau 999078, China; 4 Department of Electrical and Computer Engineering, Faculty of Science and Technology, University of Macau, Macau 999078, China

**Keywords:** calibration strip, immunochromatographic (ICG) assay, fuzzy c-means (FCM) algorithm, maximin-distance algorithm

## Abstract

With many benefits and applications, immunochromatographic (ICG) assay detection systems have been reported on a great deal. However, the existing research mainly focuses on increasing the dynamic detection range or application fields. Calibration of the detection system, which has a great influence on the detection accuracy, has not been addressed properly. In this context, this work develops a calibration strip for ICG assay photoelectric detection systems. An image of the test strip is captured by an image acquisition device, followed by performing a fuzzy c-means (FCM) clustering algorithm and maximin-distance algorithm for image segmentation. Additionally, experiments are conducted to find the best characteristic quantity. By analyzing the linear coefficient, an average value of hue (*H*) at 14 min is chosen as the characteristic quantity and the empirical formula between *H* and optical density (OD) value is established. Therefore, *H*, saturation (*S*), and value (*V*) are calculated by a number of selected OD values. Then, *H*, *S*, and *V* values are transferred to the RGB color space and a high-resolution printer is used to print the strip images on cellulose nitrate membranes. Finally, verification of the printed calibration strips is conducted by analyzing the linear correlation between OD and the spectral reflectance, which shows a good linear correlation (*R*^2^ = 98.78%).

## 1. Introduction

Lateral flow immunoassay, also known as immunochromatographic (ICG) assay, has been reported on a great deal for its several benefits—high sensitivity, ease of operation, low budget, etc. [1,2,3,4]. It utilizes antigen and antibody properties for the rapid detection of an analyte. Among the diverse labels of the antibody, colloidal gold particles are widely used [2,5,6,7,8]. Accordingly, colloidal gold-based ICG assay has been demonstrated to be potentially useful for medical diagnosis and detection of food safety, pathogen, drugs, environment, etc. [5,8,9,10,11,12,13,14]. Therefore, more and more research focuses on the applications of colloidal gold-based ICG assay from qualitative or semi–qualitative detection with the naked eye for precise quantitative detection.

Based on the operating principles and the concerned hardware of the ICG assay detection system, they can be categorized into two groups, which are image processing detection systems and photoelectric detection systems, respectively. Image processing detection systems use an image capture unit (camera or image scanner) to obtain an image of the whole test strip and performs the specific image processing algorithm to achieve the detection results. For example, Chia-Hsien et al. presents an optical inspection system based on the Taguchi method, which can achieve better linearity and decrease the standard deviation [15]. In photoelectric detection systems, a moving unit driven by a driving motor is used to scan the test strip and a photodiode is employed for photoelectric conversion. The scans can be performed very rapidly and it gets a 1-D signal along the scanning axis which results in a lower computational burden. In this context, several studies have been reported on photoelectric detection systems for ICG assays. For example, Gu et al. developed a portable fluorescence reader for the determination of C-reactive protein, which has a good sensitivity of 0.1 mg/L and linear dynamic range extended to 400 mg/L [16]. Yan et al. reports an ICG assay-based biosensor for rapid quantitative detection of *Yersinia pestis* [17]. Obviously, the existing research mainly focus on increasing the dynamic detection range or application fields. However, calibration of the detection system, which has a great influence on the detection accuracy, has not been addressed properly. Therefore, this work develops a printed calibration strip for the calibration of an ICG assay-based photoelectric detection system.

Optical density (OD) value indicates the amount of light absorbed by a solution of organic molecules on the test strip measured by a spectrophotometer, which can be used to estimate the concentration of the colloidal gold particles on the test strip. Therefore, this work was based on analyzing features of the test strip by performing an image processing algorithm, which seeks the relation of the OD value and characteristic quantity of the test strip image. According to the obtained color information of hue (*H*), saturation (*S*), and value (*V*) of the test strips, the calibration strip is printed. Further, a photoelectric detection system tests the printed calibration strip for verification. The general steps of this work are described as follows: firstly, an image of ICG assay test strip is captured by an image acquisition device, followed by noise reduction using mean and median filters. Then, a fuzzy c-means (FCM) clustering algorithm and maximin-distance algorithm are proposed for image processing in the HSV color space, which extracts a test line of the strip image. In addition, experiments with different HCG solutions and different detection times are conducted to find the best characteristic quantity. By analyzing the linear coefficient, an average value of *H* at 14 min is chosen as the characteristic quantity for the calibration test strip and the empirical formula between *H* and OD values is obtained. Therefore, *H* is predicted by a number of selected OD values and *S* and *V* are calculated. Then, *H*, *S*, and *V* are transferred to the RGB color space and a high-resolution printer is used to print the RGB image of the test strip on cellulose nitrate membranes. Finally, verification of these printed calibration strips is performed by analyzing the linear correlation between OD and the spectral reflectance of the printed calibration strips.

The rest of this paper is organized as follows: Section 2 introduces the quantitative detection system followed by methodology in Section 3. Section 4 presents the experimental results and discussions. Finally, the conclusions are drawn in Section 5.

## 2. Quantitative Detection System

### 2.1. Principle of Quantitative Detection

The Beer-Lambert Law describes the relation between the attenuation of light and the properties of the material through which the light is traveling [18]. It is the basis for the principle of quantitative detection. By definition, it describes the relationship of *A* (the absorbance of solution), *b* (thickness of medium that absorbs the incident light), and *c* (concentration of solution). According to quantum theory, when a monochromatic parallel light irradiates a uniform medium of solution, the total absorbance of the medium is the sum of absorbance of every individual object. i.e.:

(1) $$A=\sum_{i=1}^{m}\varepsilon_{i}bc_{i}$$

where $\varepsilon_{i}$ and $c_{i}$ are molarity and constant, respectively. When there is only one kind of absorbent medium, Equation (1) can be simplified as:

(2) A=εbc

In photoelectric detection systems for colloidal gold-based ICG assay, the test line on the detection strip can be regarded as a thin layer of solution with a certain thickness *b*. Therefore, Equation (2) is simplified as:

(3) $$A=k^{\prime }c$$

where $k^{\prime }=\varepsilon b$ is constant. This indicates that *A* (the absorbance of test line) is proportional to *c* (concentration of solution). Thus, when stable parallel light irradiates the test line of the strip, the darker the color of test line is, the larger *A* is, and the smaller the intensity of reflective light, whereas the lighter the color the test line is, the smaller *A* is, and the larger the intensity of reflective light. Collected by optical fiber, reflective light is focused on a photodiode which transfers optical signals into electric signals. Finally, the test strip can be quantitatively detected by analyzing the electric signals.

Conversely, in image processing detection system for colloidal gold-based ICG assay, when stable parallel light irradiates the surface of the medium, there is no light reflecting or penetrating in the ideal case, which means that all light is absorbed by the medium. Therefore, *A* is approximately equal to the integral optical density (*IOD*). *IOD* is given by:

(4) $$IOD=\sum_{i=1}^{N}OD^{\left(i\right)}=\sum_{i=1}^{N}\mathrm{lg}\frac{\phi_{0}}{\phi^{\left(i\right)}}$$

where *OD^(i)^* is the *IOD* of pixel *i*, $\phi_{0}$ is the reflective optical density of zero concentration of the solution, $\phi^{\left(i\right)}$ is the reflective optical density of pixel *i*, and *N* is the total number of image pixels. In the ideal case, both the background of the strip image and regions outside the test line are white, which indicates that all incident light is reflected. Thus, the density of incident light is equal to the density of reflected light. Additionally, for CCD or CMOS image sensors with linear photoelectric characteristics, the output current of sensors is proportional to the optical density of incident light. Hence:

(5) $$IOD=\sum_{i=1}^{N}\mathrm{lg}\frac{\phi_{0}}{\phi^{\left(i\right)}}=\sum_{i=1}^{N}\mathrm{lg}\frac{I_{0}}{I^{\left(i\right)}}=\sum_{i=1}^{N}\mathrm{lg}\frac{G_{0}}{G^{\left(i\right)}}$$

where *I^(i)^* and *G^(i)^* are the output current and gray value of pixel *i*, respectively, *I_0_* and *G_0_* are the output current and gray value of the strip background, respectively. Thus, the concentration of solution can be calculated by measuring the gray value of test line and background of the image, which indicates that the test strip can be quantitatively detected by performing specific image processing algorithms.

### 2.2. Photoelectric Detection System

**Figure 1:**
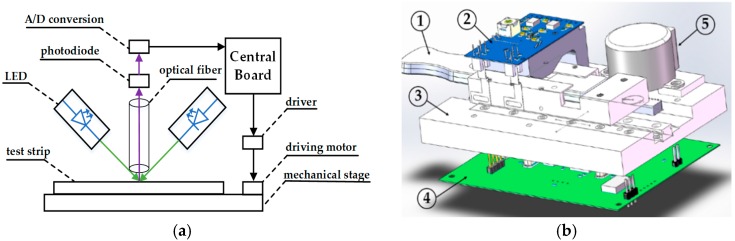
(**a**) Schematic diagram of photoelectric detection system; and (**b**) the 3D structure of the photoelectric detection system; with 1: test strip; 2: photoelectric module; 3: mechanical stage; 4: central board; and 5: driving module.

As aforementioned in Section 1, the photoelectric detection system is superior to image processing detection systems in detection speed and computational burden, which attracts many researchers. Figure 1a displays the schematic diagram of photoelectric detection system, including a mechanical module, a photoelectric module, and a central board. In the mechanical module, the test strip is placed on the mechanical stage, which is driven in and out by a driving motor. In the optical module, two LEDs irradiate light on the test strip, as indicated by green arrows. An optical fiber is placed vertically above the test strip, through which the reflective light is focused on a photodiode for photoelectric conversion, as indicated by red purple arrows. Following that, electrical signal is transferred to a digital signal by an A/D conversion unit. The central board, which is equipped with a high-performance embedded processor, controls the whole detection procedure and performs the signal processing algorithm. The 3D structure of photoelectric detection system is shown in Figure 1b, in which major components including the test strip, photoelectric module, mechanical stage, central board, and driving module.

The selection of an LED light source is based on the principle of complementary color. By analyzing the test line absorption spectrum of the colloidal gold ICG assay test strip, the maximum absorption wavelength is found at 525 nm, which is within the green light wavelength range (500~560 nm). The complementary color of green is red-purple. Therefore, according to the principle of complementary color, the test line of the strip absorbs green light and shows red-purple. In this regard, a green LED is selected to get the highest excitation energy. Furthermore, the linear working range of photoelectric sensors, the transmission characteristics of the optical fiber, as well as the parameters of the filter and amplifying circuit in the central board, have certain differences during manufacturing. They may even drift with different rules after a long time of operation, e.g., variation of the temperature and humidness, which have a great impact on detection accuracy and repeatability of the photoelectric detection system. Therefore, calibration of the detection system plays a significant role before the detection, and how to extract the property of the ICG test strips for designing the calibration strip in the next step becomes the primary problem.

## 3. Methodology

### 3.1. Image Acquisition and Processing

#### 3.1.1. Structure of Test Strip

**Figure 2:**
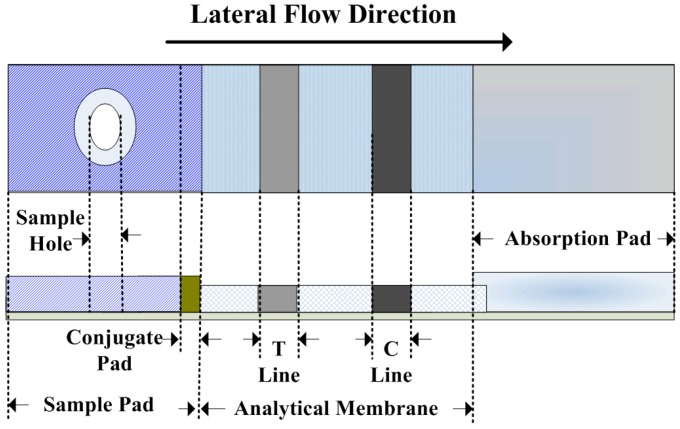
Schematic diagrams of the ICG assay test strip.

In general, an ICG assay test strip consists of three sections, including the sample pad, analytical membrane, and absorption pad, as shown in Figure 2. The sample pad includes a sample hole, through which the test samples are placed by drops. In a conjugate pad, colloidal gold nanoparticles are used as the markers for the specific target antigen. On the analytical membrane, there are a T (Test) line and C (Control) line where the antibody is placed. The T line indicates the concentration of the test sample, whereas the C line confirms the validity of the test. The absorption pad, which is located at the other end of the test strip, creates capillary action. As the test samples starts to flow from the sample pad to the absorption pad, as indicated by the arrow, immunoreaction occurs in the conjugate pad to form the conjugated particles (colloidal gold-labelled antigen-antibody complex). Then, the conjugated particles wick along the analytical membrane where another immunoreaction occurs on the T line and the C line. The rest of the particles will continue their journey until they reach the absorption pad. Finally, test results can be interpreted from the color of the T line and C line.

#### 3.1.2. Image Acquisition Device

**Figure 3:**
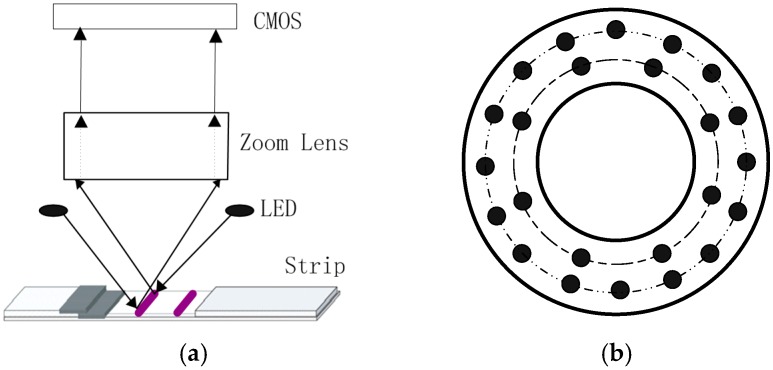
(**a**) Structure of the image acquisition device; and (**b**) structure of the light source.

The image acquisition device is mainly composed of three parts—CMOS, zoom lens, and LED light source, as shown in Figure 3a. The LED light source is designed as a cyclic structure to improve the quality of the captured image. It is placed directly above the test strip at a distance of 39.00 cm. As indicated by solid black dot in Figure 3b, there are 8 LEDs in the inner loop and 16 LEDs in the outer loop. Additionally, a 10-bit ADC CMOS image sensor is selected to capture the strip image. Between the CMOS and LED light source, there is a zoom lens to adjust the focal length. Obviously, external natural lights have a great influence on the quality of the acquired image. Therefore, this device works inside a black box to eliminate the outside interference. Finally, the acquired image is transmitted to computer by USB for further processing.

#### 3.1.3. Image Processing

The acquired image of the test strip can be segmented into three parts, which are strip shell, background, and test line part. Nevertheless, only the test line part contains the detection information. Therefore, image segmentation and test line extraction should be conducted.

Basically, RGB, HSV, and YUV are three representative color spaces which are commonly used in the image processing field [19]. However, RGB and YUV are mainly applied in raw data and coding standards, whereas HSV is more closer to human perceptions [20]. In this regard, the HSV color space is selected to perform the fuzzy c-means (FCM) clustering algorithm and maximin-distance algorithm for strip image segmentation.

The FCM clustering algorithm, which is an unsupervised clustering technique, has been widely used in biomedical image segmentation [21,22,23,24,25]. In the FCM clustering algorithm, fuzzy data is classified into a proper subset by minimizing the objective function. Assuming the given sample set is $X=\left\{x_{1},x_{2},\cdots ,x_{n}\right\}\subset R^{s}$, sample space dimension is *s*, sample amount is *n*, and *c* (*1 < c < n*) is subset amount after classifying. In this case, the FCM can be described as follows:

(6) $$J(U,V)=\sum_{i=1}^{c}\sum_{j=1}^{n}u_{ij}^{m}d_{ij}^{2}$$

where

(7) $$\sum_{i=1}^{c}u_{ij}=1,\;1\leq j\leq n$$

(8) $$\sum_{j=1}^{n}u_{ij}>0,\;1\leq i\leq c$$

(9) $$u_{ij}\geq 0,1\leq i\leq c,1\leq j\leq n$$

In Equation (6) to Equation (9), *m* (*m* > 1) is the fuzzy parameter, $U=u_{ij}$ is a c×n fuzzy partition matrix, *u_ij_* indicates the membership value of *x_j_* that belongs to class *i*, $V=\left[v_{1},v_{2},\cdots ,v_{c}\right]$ is a s×c matrix which is composed of *c* clustering center vectors, and $d_{ij}=\left\|x_{j}-v_{i}\right\|$ is the distance between sample point *x_j_* and center point *v_i_*. Therefore, the fuzzy clustering algorithm is evolved into the optimization of the restrained argument of (U,V). Then, the iterative equation is acquired by the necessary conditions of the extreme point.

(10) $$v_{i}=\frac{\sum_{j=1}^{n}u_{ij}^{m}x_{j}}{\sum_{j=1}^{n}u_{ij}^{m}},\;i=1,2,\cdots ,c$$

Assuming $I_{j}=\left\{\left(i,j\right)|x_{j}=v_{i},1\leq i\leq c\right\}$, if $I_{j}=\emptyset$, then

(11) $$u_{ij}={\left[{\sum_{r=1}^{c}\left(\frac{d_{ij}}{d_{rj}}\right)}^{\frac{2}{m-1}}\right]}^{-1}i=1,2,\cdots ,c;j=1,2,\cdots ,n$$

If $I_{j}\neq \emptyset$, then *u_ij_* is the arbitrary non-negative real number that satisfies the following condition:

(12) $$\sum_{i=1}^{c}u_{ij}=1;u_{ij}=0,d_{ij}\neq 0$$

The equation of membership degree shows the mapping relationship from point to set and the membership degree can be updated by Equation (13). Now, the implementation steps of the FCM algorithm are: firstly, initialize the clustering center or membership degree matrix; then, Equations (10) and (13) are iterated until the inequality (14) is satisfied. Specific steps of the FCM algorithm are explained in Table 1.

(13) $$u_{ij}=\{\begin{array}{c} {\left[{\sum_{r=1}^{c}\left(\frac{d_{ij}}{d_{rj}}\right)}^{\frac{2}{m-1}}\right]}^{-1},I_{j}=\emptyset \\ \frac{1}{\left|I_{j}\right|},I_{j}\neq \emptyset ,i\in I_{j}\\ \begin{array}{l} 0,I_{j}\neq \emptyset ,i\notin I_{j} \end{array} \end{array}$$

(14) $$\left\|V^{\left(k\right)}-V^{\left(k-1\right)}\right\|\leq \varepsilon ,k\geq 1$$

A maximin-distance algorithm is a simple heuristic procedure which can be used in initializing the cluster center in the FCM algorithm to assure stability of the result and to avoid random initialization [26,27,28,29]. The specific steps of algorithm is described in Table 2.

### 3.2. Development and Verification of the Calibration Strip

**Figure 4:**
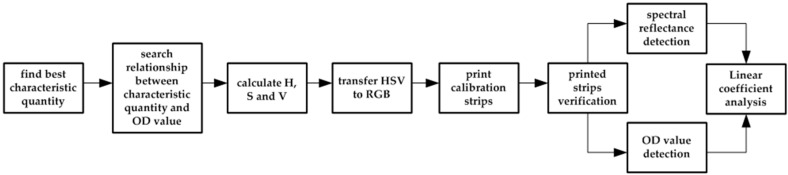
Development and verification steps of the calibration strip.

Development and verification steps of the calibration strip are shown Figure 4. The most important part of the development of the calibration strip is searching the relationship between the OD value and characteristic quantities of the test strip image. However, in colloidal gold-based ICG assay, the binding of antigen and antibody is a dynamic process. Therefore, color depth of the test line of the strip is changing over time because of capillarity and siphon action, i.e., there is no exact end for the chromatographic process. Certainly, selection of the detection time has a great impact on the detection accuracy. In this regard, experiments with different concentrations of solutions and different detection times are conducted to find the best characteristic quantities of the strip image. By analyzing the linear coefficient, the empirical formula between OD value and characteristic quantities of the strip image is obtained, by which *H*, *S*, and *V* are calculated. In order to print the strip image with a high-resolution printer, the obtained *H*, *S*, and *V* values are transferred to the RGB color space. Finally, the printed calibration strip is produced on cellulose nitrate membranes.

As aforementioned in Section 2, the principle of quantitative detection is based on the Beer-Lambert Law, which indicates that the color depth of the test line in different concentrations is linear with the absorbance of the test line. Therefore, verification of the printed calibration strips can be evaluated by the absorption spectral peak value of the test line. In this context, verification of the calibration strip is conducted by analyzing the linear correlation between the OD value and the spectral reflectance of the printed calibration strip.

## 4. Experimental Results and Discussion

### 4.1. Experiment of Test Line Extraction

**Figure 5:**
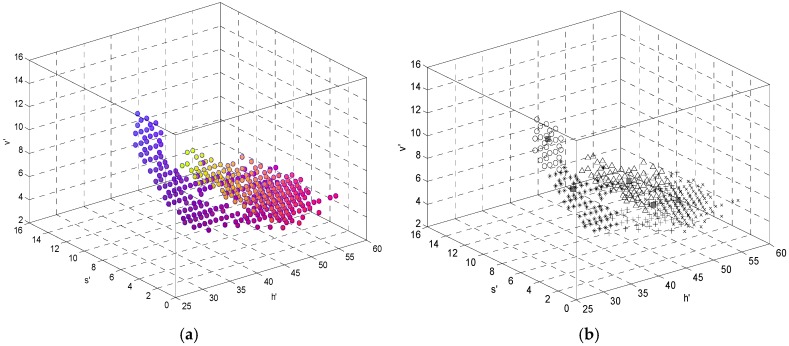
(**a**) Three-dimensional histogram of h′s′v′; and (**b**) the result of FCM clustering. ■ is the position of the clustering center and the other five symbols are five different clusterings.

Basically, there is mainly Gaussian noise and impulse noise in the original image. Thus, a mean filter and median filter are performed to reduce noise before implementing the test line extraction algorithm. In order to improve processing efficiency, *H*, *S*, and *V* are divided by 6°, 0.0625, and 0.0625, respectively, as described in Equation (15). This reduces the image dimensions without losing the main features of the image [30,31]. The three-dimensional *h’ s’ v’* histogram of the test strip image (concentration is 350 mIU/mL) is shown in Figure 5a.

(15) $$h\prime =H/6^{o};s\prime =S/0.0625;v\prime =V/0.0625$$

**Figure 6:**
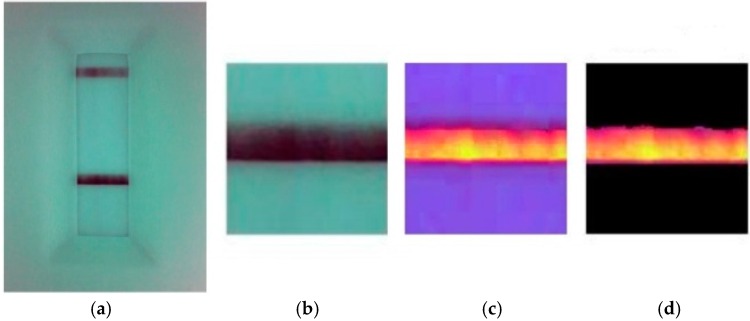
Process of image segmentation. (**a**) Original image; (**b**) the segmented test line; (**c**) HSV image with adjusted brightness; and (**d**) the result of test line segmentation.

In this histogram, the largest number of pixels is selected as the first clustering center. Based on the aforementioned maximin-distance algorithm, the clustering number *c* and clustering center *V*^(^°^)^ are determined. Then, a FCM algorithm is implemented on the HSV color space to segment the strip image. The clustering result is shown in Figure 5b. Without insufficient or over segmentation of the test line, the segmentation algorithm has high performance, as shown in Figure 6, where (a) is the original image, (b) is the segmented test line, (c) is the HSV image with adjusted brightness, and (d) is the resulting test line segmentation.

### 4.2. Development of the Calibration Strip

**Figure 7:**
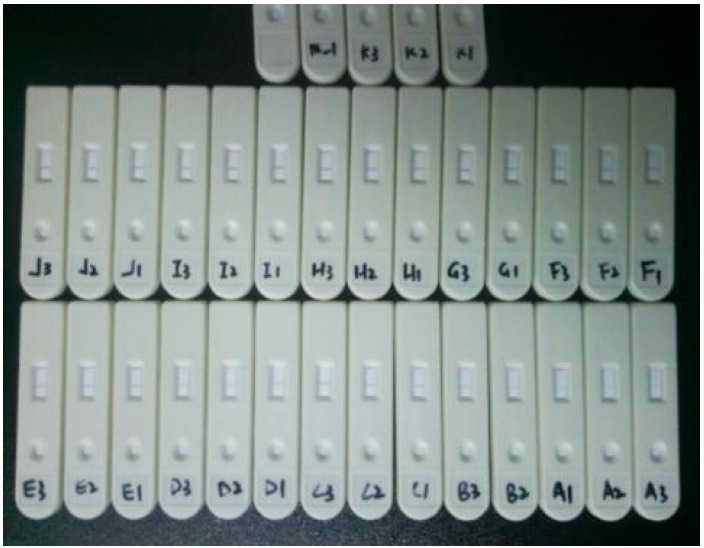
Test strips for colloidal gold-based ICG assay.

HCG solution, which has been used in preliminary selection of Down’s syndrome and the diagnosis of early pregnancy, or eccyesis, is chosen as the reagent for extracting characteristic quantities. In addition, test strips are selected from diagnostic kits (Xiamen Boson Biotech Co., Ltd., Xiamen, China) for rapid quantitative determination of human choriogonadotropin (β-HCG) in the same patch and specification. The detection sensitivity of the strip is 10 mIU/mL. Additionally, the concentration of diluents involves 10, 50, 100, 150, 200, 250, 300, 350, 400, 450, 500 mIU/mL. Generally, the best detection time is 10 min~18 min after the sample solution is dropped into the sample pad. Therefore, the image acquisition device captures the strip image at 10 min, 12 min, 14 min, 16 min, and 18 min. Then, the OD value of the acquired strip image is immediately measured by a quantitative detection system for ICG assay (SWP-SC-2). To reduce errors, every concentration of diluent is repeatedly measured three times. The test strips are shown in Figure 7.

As the light source of image acquisition device is not exactly stable, which results in the instability of *V* in the HSV color space, four characteristic quantities of the strip image are selected from *H* and *S*, which are one- or two-dimensional mean values of H, 360×H, 10×H×S, and $\sqrt{H^{2}+{\left(360\times S\right)}^{2}}$. The linear coefficient between these characteristic quantities and concentrations of HCG, and OD values, are shown in Table 3 and Table 4, respectively. As shown, an average value of *H* at 14 min has the maximum linear coefficient (95.24%) with the concentration of HCG. Further, the linear coefficient between an average value of *H* at 14 min and OD value is over 98%. Thus, an average value of *H* at 14 min is chosen as the characteristic quantity for the calibration test strip.

Now, the empirical formula between *H* and OD value is obtained by:

(16) H=10.153×OD+262.427

From Equation (16), the mean value of *H* can be calculated from the OD value. Selecting a number of OD values (1.5, 3, 4, 5, 6, 7, 8, 9, 9.5), the predicted mean values of *H* are shown in Table 5.

**Figure 8:**
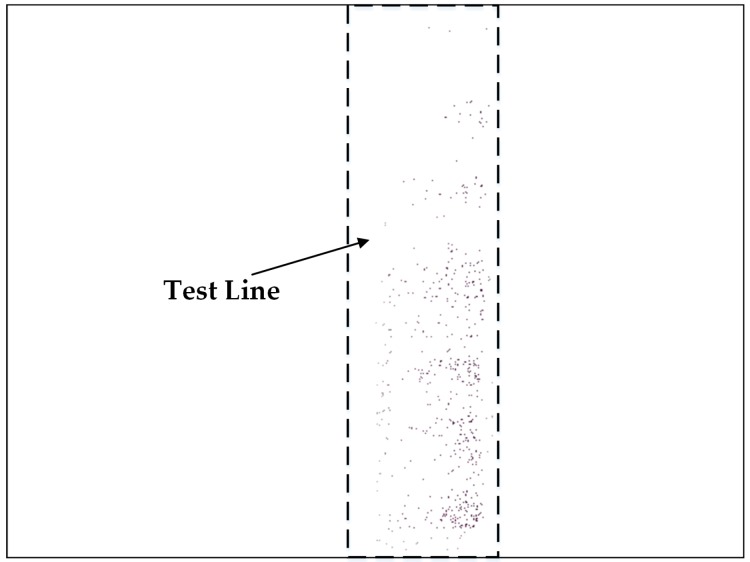
Valid points of the segmented image.

In order to determine *S* and *V*, the pixels of *pix_ij_* that satisfied the inequality ($H-0.25<H_{pix_{ij}}\leq H+0.25$) are regarded as valid points. For example, if *H* = 303.039, the valid points of the segmented strip image is shown in Figure 8. Hence, *S* and *V* is calculated by Equation (17).

(17) $$S=\sum S_{pix_{ij}}/N;\;V=\sum V_{pix_{ij}}/N$$

**Figure 9:**
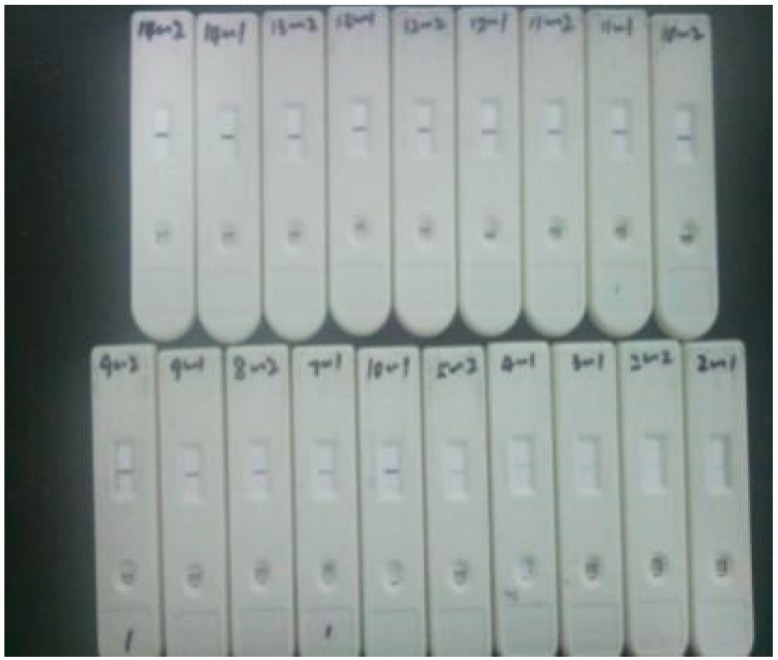
Printed calibration test strips.

Then, the obtained *H*, *S*, and *V* values are transferred to the RGB color space, which is more suitable for printing. A high-resolution color inkjet printer is used to print the RGB image on cellulose nitrate membranes. The printed calibration test strips are shown in Figure 9.

### 4.3. Verification of the Calibration Strip

**Figure 10:**
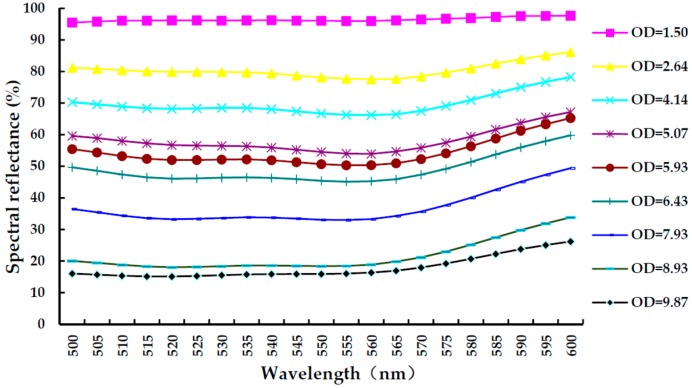
The spectral reflectance of test line of the printed calibration strips.

**Figure 11:**
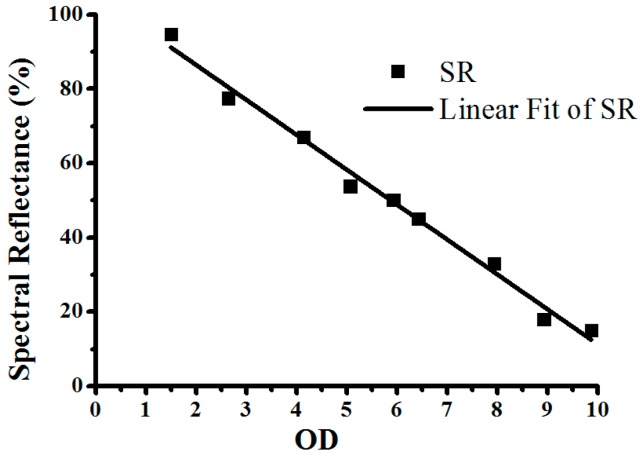
Linear fit of the spectral reflectance.

In the verification of the printed calibration strips, OD values for different concentrations of the test strips (the same test kit as aforementioned in Section 4.2) is read by a quantitative detection system for ICG assay (SWP-SC-2), which are 1.50, 2.64, 4.14, 5.07, 5.93, 6.43, 7.93, 8.93, and 9.87. Additionally, an OL750 Automated Spectroradiometric Measurement System (Optronic Laboratories Inc., Orlando, Florida, America) is used to measure the spectral reflectance of test line as displayed in Figure 10, in which the horizontal and vertical direction refer to wavelength (500~600 nm, increase by 5 nm) and spectral reflectance, respectively. Therefore, the average spectral reflectance of the test line is calculated, as listed in Table 6, where first column is the OD value and second column is the average spectral reflectance of the test line (wavelength from 500 to 600 nm). The linear fit of the average spectral reflectance and OD is shown in Figure 11, which demonstrates that OD of the printed calibration strips has a good linear correlation with the spectral reflectance (*R*^2^ = 98.78%). In conclusion, the developed printed calibration strip is effective for ICG assay photoelectric detection systems.

## 5. Conclusions

This work develops a calibration strip for immunochromatographic (ICG) assay photoelectric detection systems. An image of the test strip is captured by an image acquisition device. Mean and median filters are used to reduce noise in the acquired image. Without insufficient or over segmentation, the proposed FCM algorithm and maximin-distance algorithm has a good performance on extraction of the test line. Further, experiments with different HCG solution and different detection times are conducted to find the best characteristic quantity, which indicates that the average value of *H* at 14 min has the best linear coefficient with a concentration of HCG (95.24%) and OD value (98.12%). Therefore, the empirical formula between *H* and OD (optical density) values is established, by which *H*, *S*, and *V* are calculated. Additionally, *H*, *S*, and *V* values are transferred to the RGB color space and a high-resolution printer is used to print the RGB images on cellulose nitrate membranes. Finally, OD and the spectral reflectance of the printed calibration strips are analyzed with a good linear correlation (*R*^2^ = 98.78%), which indicates that the developed printed calibration strip is effective for the calibration of the ICG assay detection system.

## Figures and Tables

**Table 1:** Specific steps of the FCM clustering algorithm.

**Step 1:** Set clustering number *c* and fuzzy exponent *m*; initialize center of clustering *V*^(^°^)^; set convergence accuracy ε and iteration times *k*.
**Step 2:** Calculate *V*^(^°^)^ according to Equation (13).
**Step 3:** Let *k = k* + 1, calculate *V*^(*k* + 1)^ according to Equation (10).
**Step 4:** Repeat Steps 2 and 3 until inequality (14) is satisfied.

**Table 2:** Procedure of the maximin-distance algorithm.

**Step 1:** Assuming the dataset *X* is composed of n vectors, i.e., $X=\left\{x_{1},x_{2},\ldots x_{n}\right\}$; aibitrarily select one vector (*x_1_*) from the dataset as the first clustering *v_1_*, e.g., *v_1_* = *x_1_*.
**Step 2:** Calculate the distances between *v_1_* and all other points in the set, find the point with the largest distance and set as *v_2_*.
**Step 3:** Calculate the distances between the remaining vectors of *X* and the known clustering centers, and choose the minimum distances as a group. Then, select the maximum distance in this group. If the maximum is larger than the given threshold $m|Z_{2}-Z_{1}|$, this point will be set as a new clustering center. Generally, 0.5≤m≤1.
**Step 4:** Repeat Step 3 until the acquired maximum distance does not satisfy the condition of creating a new center or the value of the clustering center reaches the desired number.

**Table 3:** Linear coefficient between characteristic quantities and concentration of HCG (%).

	Time (min)	10	12	14	16	18
CQ						
H		95.15	95.04	95.24	94.61	94.22
360×H		79.58	84.70	88.34	89.45	91.35
10×H×S		84.57	87.54	90.21	90.01	91.88
$\sqrt{H^{2}+{\left(360\times S\right)}^{2}}$		79.84	84.81	88.41	89.50	90.82

**Table 4:** Linear coefficient between characteristic quantities and OD value (%).

	Time (min)	10	12	14	16	18
CQ						
H		98.02	98.31	98.12	98.11	97.93
360×H		81.33	87.87	90.91	92.18	93.11
10×H×S		86.77	90.92	93.02	93.90	94.85
$\sqrt{H^{2}+{\left(360\times S\right)}^{2}}$		81.64	88.02	91.03	92.27	93.56

**Table 5:** The predicted mean values of *H*.

OD	*H*
1.5	277.657
3	292.886
4	303.039
5	313.192
6	323.345
7	333.498
8	343.651
9	353.804
9.5	358.881

**Table 6:** The calculated average spectral reflectance of the test line.

OD	SR
1.50	94.83
2.64	77.49
4.14	67.05
5.07	53.87
5.93	50.21
6.43	45.06
7.93	33.02
8.93	18.05
9.87	15.09

## References

[1] Cho Y.-J., Lee D.-H., Kim D.-O., Min W.-K., Bong K.-T., Lee G.-G., Seo J.-H. (2005). Production of a monoclonal antibody against ochratoxin A and its application to immunochromatographic assay. J. Agric. Food Chem..

[2] Shyu R.-H., Shyu H.-F., Liu H.-W., Tang S.-S. (2002). Colloidal gold-based immunochromatographic assay for detection of ricin. Toxicon.

[3] Sun X., Zhao X., Tang J., Gu X., Zhou J., Chu F.S. (2006). Development of an immunochromatographic assay for detection of aflatoxin B_1_ in foods. Food Control.

[4] Yang W., Li X.-B., Liu G.-W., Zhang B.-B., Zhang Y., Kong T., Tang J.-J., Li D.-N., Wang Z. (2011). A colloidal gold probe-based silver enhancement immunochromatographic assay for the rapid detection of abrin-a. Biosens. Bioelectron..

[5] Chiao D.-J., Shyu R.-H., Hu C.-S., Chiang H.-Y., Tang S.-S. (2004). Colloidal gold-based immunochromatographic assay for detection of botulinum neurotoxin type B. J. Chromatogr. B.

[6] Shim W., Yang Z., Kim J., Kim J., Kang S., Woo G., Chung Y., Eremin S.A., Chung D. (2007). Development of immunochromatography strip-test using nanocolloidal gold-antibody probe for the rapid detection of aflatoxin B1 in grain and feed samples. J. Microbiol. Biotechnol..

[7] Shim W.-B., Yang Z.-Y., Kim J.-Y., Choi J.-G., Je J.-H., Kang S.-J., Kolosova A.Y., Eremin S.A., Chung D.-H. (2006). Immunochromatography using colloidal gold-antibody probe for the detection of atrazine in water samples. J. Agric. Food Chem..

[8] Zhou Y., Pan F.-G., Li Y.-S., Zhang Y.-Y., Zhang J.-H., Lu S.-Y., Ren H.-L., Liu Z.-S. (2009). Colloidal gold probe-based immunochromatographic assay for the rapid detection of brevetoxins in fishery product samples. Biosens. Bioelectron..

[9] Guo Y.-R., Liu S.-Y., Gui W.-J., Zhu G.-N. (2009). Gold immunochromatographic assay for simultaneous detection of carbofuran and triazophos in water samples. Anal. Biochem..

[10] Ju Y., Hao H.-J., Xiong G.-H., Geng H.-R., Zheng Y.-L., Wang J., Cao Y., Yang Y.-H., Cai X.-H., Jiang Y.-Q. (2010). Development of colloidal gold-based immunochromatographic assay for rapid detection of Streptococcus suis serotype 2. Vet. Immunol. Immunopathol..

[11] Kumar R., Singh C.K., Kamle S., Sinha R.P., Bhatnagar R.K., Kachru D.N. (2010). Development of nanocolloidal gold based immunochromatographic assay for rapid detection of transgenic vegetative insecticidal protein in genetically modified crops. Food Chem..

[12] Meng K., Sun W., Zhao P., Zhang L., Cai D., Cheng Z., Guo H., Liu J., Yang D., Wang S. (2014). Development of colloidal gold-based immunochromatographic assay for rapid detection of Mycoplasma suis in porcine plasma. Biosens. Bioelectron..

[13] Shyu R.-H., Tang S.-S., Chiao D.-J., Hung Y.-W. (2010). Gold nanoparticle-based lateral flow assay for detection of staphylococcal enterotoxin B. Food Chem..

[14] Sheng W., Li Y., Xu X., Yuan M., Wang S. (2011). Enzyme-linked immunosorbent assay and colloidal gold-based immunochromatographic assay for several (fluoro) quinolones in milk. Microchim. Acta.

[15] Yeh C.-H., Zhao Z.-Q., Shen P.-L., Lin Y.-C. (2014). Optimization of an Optical Inspection System Based on the Taguchi Method for Quantitative Analysis of Point-of-Care Testing. Sensors.

[16] Gu Y., Yang Y., Zhang J., Ge S., Tang Z., Qiu X. (2014). Point-of-care test for C-reactive protein by a fluorescence-based lateral flow immunoassay. Instrum. Sci. Technol..

[17] Yan Z., Zhou L., Zhao Y., Wang J., Huang L., Hu K., Liu H., Wang H., Guo Z., Song Y. (2006). Rapid quantitative detection of Yersinia pestis by lateral-flow immunoassay and up-converting phosphor technology-based biosensor. Sens. Actuators B Chem..

[18] Vieira G.P., Perdigão S.R., Fiore M.F., Reis B.F. (2012). Development of a high sensitive automatic setup for screening of microcystins in surface waters by employing a LED-based photometric detector. Sens. Actuators B Chem..

[19] Phung S.L., Bouzerdoum A., Chai D. (2005). Skin segmentation using color pixel classification: Analysis and comparison. IEEE Trans. Pattern Anal. Mach. Intell..

[20] Sural S., Qian G., Pramanik S. Segmentation and histogram generation using the HSV color space for image retrieval. Proceedings of the 2002 IEEE International Conference on Image Processing.

[21] Akbari H., Kalkhoran H.M., Fatemizadeh E. A robust FCM algorithm for image segmentation based on spatial information and Total Variation. Proceedings of the 9th IEEE Iranian Conference on Machine Vision and Image Processing (MVIP).

[22] Lan T., Xiao Z., Hu C., Ding Y., Qin Z. MRI brain image segmentation based on Kerneled FCM algorithm and using image filtering method. Proceedings of the 2014 IEEE International Conference on Audio, Language and Image Processing (ICALIP).

[23] Song X., Li G., Luo L. Optimizing FCM for segmentation of image using Gbest-guided artificial bee colony algorithm. Proceedings of the 2015 11th IEEE International Conference on Natural Computation (ICNC).

[24] Tang D.-Y., Yang J., Huang Y.-S. Double weighted FCM algorithm for color image segmentation. Proceedings of the 2012 IEEE International Conference on Machine Learning and Cybernetics (ICMLC).

[25] Wang P., Wang H. A modified FCM algorithm for MRI brain image segmentation. Proceedings of the 2008 IEEE International Seminar on Future BioMedical Information Engineering (FBIE’08).

[26] Dimitriadou E., Barth M., Windischberger C., Hornik K., Moser E. (2004). A quantitative comparison of functional MRI cluster analysis. Artif. Intell. Med..

[27] Jarmasz M., Somorjai R.L. (2002). Exploring regions of interest with cluster analysis (EROICA) using a spectral peak statistic for selecting and testing the significance of fMRI activation time-series. Artif. Intell. Med..

[28] Pizzi N.J., Vivanco R.A., Somorjai R.L. (2001). EvIdent™: A functional magnetic resonance image analysis system. Artif. Intell. Med..

[29] Somorjai R.L. (2002). Exploratory data analysis in functional neuroimaging. Artif. Intell. Med..

[30] Cheng B., Zhuo L., Zhang J. Comparative Study on Dimensionality Reduction in Large-Scale Image Retrieval. Proceedings of the 2013 IEEE International Symposium on Multimedia (ISM).

[31] Lei L., Wang X., Yang B., Peng J. Image dimensionality reduction based on the HSV feature. Proceedings of the 2010 9th IEEE International Conference on Cognitive Informatics (ICCI).

